# BCLX_L_ gene therapy moderates neuropathology in the DBA/2J mouse model of inherited glaucoma

**DOI:** 10.1038/s41419-021-04068-x

**Published:** 2021-08-10

**Authors:** Ryan J. Donahue, Rachel L. Fehrman, Jenna R. Gustafson, Robert W. Nickells

**Affiliations:** 1 Department of Ophthalmology and Visual Sciences, University of Wisconsin–Madison, Madison, WI USA; 2 Cellular and Molecular Pathology Graduate Program, University of Wisconsin–Madison, Madison, WI USA; 3 Department of Cell Biology, Neurobiology and Anatomy, Medical College of Wisconsin, Milwaukee, WI USA; 4 Department of Neurobiology and Anatomy, Wake Forest School of Medicine, Winston-Salem, NC USA; 5 McPherson Eye Research Institute, University of Wisconsin–Madison, Madison, WI USA

**Keywords:** Cell death in the nervous system, Neurodegeneration

## Abstract

Axonal degeneration of retinal ganglion cells (RGCs) causes blindness in glaucoma. Currently, there are no therapies that target axons to prevent them from degenerating. Activation of the BAX protein has been shown to be the determining step in the intrinsic apoptotic pathway that causes RGCs to die in glaucoma. A putative role for BAX in axonal degeneration is less well elucidated. BCLX_L_ (BCL2L1) is the primary antagonist of BAX in RGCs. We developed a mCherry-BCLX_L_ fusion protein, which prevented BAX recruitment and activation to the mitochondria in tissue culture cells exposed to staurosporine. This fusion protein was then packaged into adeno-associated virus serotype 2, which was used to transduce RGCs after intravitreal injection and force its overexpression. Transduced RGCs express mCherry-BCLX_L_ throughout their somas and axons along the entire optic tract. In a model of acute optic nerve crush, the transgene prevented the recruitment of a GFP-BAX fusion protein to mitochondria and provided long-term somal protection up to 12 weeks post injury. To test the efficacy in glaucoma, DBA/2J mice were transduced at 5 months of age, just prior to the time they begin to exhibit ocular hypertension. Gene therapy with mCherry-BCLX_L_ did not affect the longitudinal history of intraocular pressure elevation compared to naive mice but did robustly attenuate both RGC soma pathology and axonal degeneration in the optic nerve at both 10.5 and 12 months of age. BCLX_L_ gene therapy is a promising candidate for glaucoma therapy.

## Introduction

In glaucoma, blindness is caused by degeneration of retinal ganglion cells (RGCs), neurons that project axons from the retina to the brain. The most prominent risk factor for glaucoma is elevated intraocular pressure (IOP) [1], and currently, lowering IOP is the only viable treatment for this disease. While this treatment is often able to slow the progression of neurodegeneration [2], there is wide-spread acknowledgement that a therapeutic that directly targets the RGCs and their axons would significantly augment IOP lowering therapies.

Evidence suggests that RGC axons are injured early in glaucoma and can degenerate independently of the soma [3]. Mechanisms of axon degeneration have been an area of intense study for the past two decades. There is evidence for axon degeneration pathways mediated independently by SARM1 [3–5] and BAX [6–9]. In RGCs, BAX is essential for the execution of the caspase-dependent, intrinsic apoptotic program [6, 10, 11], but its role in glaucomatous axon degeneration is controversial [3, 5].

*Bcl2l1* (hereafter designated as *BclX*_*L*_) is an anti-apoptotic member of the *Bcl2* gene family, and in central nervous system neurons is the primary antagonist of BAX [12]. BCLX_L_ exists primarily at the mitochondria and inhibits BAX activation by preventing BAX from accumulating on the mitochondrial outer membrane [13]. Overexpression of BCLX_L_ protects neurons from death following trophic factor withdrawal and ischemia–reperfusion [14–16]. In RGCs, increasing the intracellular concentration of BCLX_L_ prevents the degeneration of somas and proximal axon segments after axotomy [17, 18].

The DBA/2J mouse is a widely used model of glaucoma that develops spontaneous, asynchronous elevation of IOP around 6 months of age that persists until the mice are nearly a year old [19–21]. This causes degeneration of RGCs and makes the DBA/2J mouse a useful model for studying glaucomatous neurodegeneration. Experimentally, the ages of 10.5 and 12 months are frequently used for assessing degeneration. Some therapeutic approaches are protective exclusively at 10.5 months of age [6, 22], whereas others are protective at both 10.5 and 12 months of age [23–25]. The differential effect of therapies is evidence that multiple axon degenerative pathways contribute to glaucomatous neurodegeneration. Intriguingly, deletion of the *Bax* gene conferred protection only at 10.5 months of age but deletion of *Bim*, a protein that is important for activating BAX in RGCs, protected optic nerves (ONs) at both 10.5 and 12 months of age, suggesting that the BCL2 family may mediate axon degeneration independent of BAX activity in RGCs [6, 24]. Notably, both complete *Bax* and *Bim* deletion also affected the elevation of IOP, which may have contributed to their protective effect.

Gene therapy is a clinically relevant therapeutic paradigm for treating retinal disease [26]. The premise of gene therapy is to deliver a therapeutic gene to a susceptible population of cells, generally via a viral vector such as a recombinant adeno-associated virus (AAV). AAVs have low immunogenicity, are replication deficient, and are not known to cause any human disease [27]. In mice, intravitreal delivery of AAV serotype 2 (AAV2) of a sufficient titer can transduce around 85% of RGCs [28].

Gene therapy targeted directly to RGCs to prevent BAX activation in a glaucoma model has never been attempted. This approach is clinically relevant and can be performed in wild-type mice that do not have the developmental abnormalities associated with *Bax* deletion [29]. A previous study investigated BCLX_L_ gene therapy in a model of ON axotomy and achieved significant albeit transient protection of RGCs [18]. The transient nature of the protection may have been due to loss of expression of the transgene, which was expressed using a neuron-specific promoter. Neuron-specific gene expression is rapidly silenced in RGCs following ON injury [30]. Loss of expression can be circumvented by using the *Pgk* promoter, whose expression is sustained in damaged RGCs [31].

We hypothesized that AAV2-mediated BCLX_L_ gene therapy would prevent RGC degeneration in the DBA/2J mouse model of glaucoma. Overexpression of mCherry-BCLX_L_ protected both somas and axons from glaucomatous neurodegeneration. This result demonstrates a role for the BCL2 family in glaucomatous RGC somal and axonal degeneration.

## Methods

### Cloning mCherry-BclX_L_

Moloney Murine Leukemia Virus Reverse Transcriptase (Promega, Madison, WI) and Oligo(dT)_15_ Primer (Promega) were used to make cDNA from total RNA isolated from BALB/c mouse brain tissue. Polymerase chain reaction (PCR) was used to amplify the coding region of the *BclX*_*L*_ transcript from the cDNA using the following primers 5’-AAATGTCTCAGAGCAACCGGGAGCTG–3’ and 5’-CAGTGTCTGGTCACTTCCGACTGAAGAG-3’. This PCR product was ligated into the pGemT vector using T4 DNA Ligase (Promega). *BclX*_*L*_ was amplified with XhoI and HindIII restriction sites using the following primers 5’-TGGCCGGCTCGAGAAATGTCTCAG-3’ and 5’-GATTCAGTAAGCTTTCACTTCCGACTGAAG-3’. This PCR product was digested with XhoI and HindIII (Promega) and ligated into a pmCherry-C1 plasmid (Clontech, Mountain View, CA), with *BclX*_*L*_ placed downstream of mCherry. Sequencing was performed to verify that *BclX*_*L*_ had the proper sequence and was in frame with mCherry.

### Validation of mCherry-BCLX_L_ in vitro

D407 cells were grown in Dulbecco’s modified Eagle media (DMEM) (Corning, Corning, NY) containing 3% (V/V) fetal bovine serum (Atlanta Biologicals, Norcross, GA) and 1% penicillin–streptomycin (V/V) (Thermo Fisher Scientific, Waltham, MA). The GFP-BAX and mitoBFP constructs have been previously described [32, 33].

D407 cells were nucleofected using a Lonza 4D nucleofector (Lonza, Basel, Switzerland) and treated 24 h later with 1 µM staurosporine (STS) (Alfa Aesar, Ward Hill, MA). Live cell imaging was performed using an Andor Revolution XD spinning disc confocal microscope (Andor, Belfast, UK) in a 37 °C imaging chamber. Images were taken every 3 min for 3.5 h during the experiment. Image analysis was performed using the Imaris 9.2.1 software (Oxford Instruments, Abingdon, UK). The significant differences in the distribution between groups were assessed using *χ*^2^ tests.

### Viral packaging

The mCherry-BclX_L_ construct was digested using NheI and ApaI and ligated into a bridge vector created from AAV-*Pgk*-Cre (a gift from Patrick Aebischer, Addgene plasmid #24593) where the Cre coding region was replaced with a multiple cloning site containing unique NheI and ApaI sites.

Viral packaging was performed by the University of North Carolina – Chapel Hill, Vector Core. *AAV2-Pgk-mCherry-BclX*_*L*_ and *AAV2-Pgk-GFP-Bax* had titers of 1.7 × 10^13^ and 2.3 × 10^12^ viral genomes/mL, respectively.

### Mouse housing and ethics

Mice were handled in accordance with the Association for Research in Vision and Ophthalmology’s Statement for the Use of Animals in Ophthalmic and Vision Research. The research protocol was approved by the Institutional Animal Care and Use Committee of the University of Wisconsin – Madison. Mice were kept in microisolator cages on a 12-h light/dark cycle and fed a 4% fat diet. Sample sizes were estimated using power calculations (0.8, 20% effective difference) with historical variance estimates for the type of assay used. All data shown used sample sizes that exceeded the “*N*” estimated by power calculations. Animals were randomly distributed into treatment groups with effort to balance male to female ratios. For DBA/2J mouse experiments, the male:female ratio is specifically indicated for each cohort in the figure legends. Any animals that exhibited distress after inclusion in a cohort were removed from the study.

### Optic nerve crush (ONC) and intravitreal injections

For all surgeries, mice were anesthetized with 16 mg/mL ketamine and 1.5 mg/mL xylazine. Eyes were anesthetized with 0.5% proparacaine hydrochloride. Postoperative discomfort was alleviated with 0.03 mg/mL buprenorphine.

Intravitreal injection of virus or 1% cholera toxin subunit B (Alexa-488) (CTB-488; Thermo Fisher) was performed as previously described [30]. For experiments requiring injection of multiple viruses, titers were equalized by mixing the viruses followed by a single injection. Four weeks were allowed for viral genome replication and transgene expression. ONC was performed as previously described [34] using C57BL/6J mice.

### DBA/2J experiments and IOP measurements using rebound tonometry

Ten-week-old DBA/2J mice were bilaterally injected with *AAV2-Pgk-mCherry-BCLX*_*L*_. An additional cohort of 10 uninjected DBA/2J mice was used as a naive control. Each cohort contained 5 male and 5 female mice. IOPs were measured every 2 weeks between 9 and 11 a.m. using a Tonolab rebound tonometer (Icare, Finland) as previously described [35]. Eyes that developed corneal abnormalities were excluded. Longitudinal IOP history was analyzed using Generalized Estimating Equation regression modeling (R, v4.0.5).

For experiments measuring ON degeneration, DBA/2J mice between 4 and 5 or 7 months of age were bilaterally transduced with *AAV2-Pgk-mCherry-BclX*_*L*_. Naive mice received no injection. Each cohort of glaucomatous mice contained at least 49 ONs and 29 retinas. We did not use a control viral injection because other studies have found no effect on IOP progression or protection of RGCs by control AAVs [35, 36]. Because glaucomatous disease progresses asymmetrically in the eyes of DBA/2J mice [21], each eye/retina/ON was considered as an independent variable.

### Euthanasia, tissue removal, and fixation

Mice were euthanized with pentobarbital sodium and phenytoin sodium (Virbac, Westlake, TX) followed by cervical dislocation. Eyes were enucleated with a proximal piece of the ON attached and placed in 4% paraformaldehyde (PFA) in phosphate-buffered saline (PBS, 100 mM phosphate buffer with 150 mM NaCl, pH 7.4) for 1 h. For ONC experiments, mice were euthanized 1 week after ONC. For glaucoma studies, mice were euthanized at 10.5 or 12 months of age. Young DBA/2J cohorts consisted of mice <4 months of age.

### Frozen sectioning and staining of whole-eye sections

After fixation, a brief PBS washed was performed. Samples, including both retinas and ONs, were equilibrated in 30% sucrose in PBS overnight at 4 °C. Samples were then frozen on dry ice in plastic molds using O.C.T. Compound (Scigen, Paramount, CA). In all, 8 µm sections were obtained from the Translational Initiatives in Pathology (TRIP) lab at the University of Wisconsin – Madison.

Immunofluorescence was performed as previously described [30]. Mouse anti-BRN3A monoclonal antibody (MAB1585) (EMD Millipore, Temecula, CA) was used at a concentration of 1:50. Rabbit anti-mCherry polyclonal antibody (ab167453) (Abcam, Cambridge, UK) was used at a concentration of 1:500. The anti-mCherry antibody was only used to enhance the signal of the sections that were used to assess transduction efficiency. All other samples showed endogenous mCherry signal. Rabbit anti-calbindin antibody (SWANT, Marly, Switzerland) was used at a concentration of 1:1000. Goat anti-mouse immunoglobulin G (IgG) fluorescein isothiocyanate conjugated to FITC or Alexa488 and goat anti-rabbit IgG conjugated to Texas Red, Alexa594 or Alexa488 (Jackson Immunoresearch, West Grove, PA) were used at a concentration of 1:1000.

Transduction efficiency was calculated as the percentage of 4,6-diamidino-2-phenylindole (DAPI)-positive nuclei that had mCherry-BCLX_L_ labeling in the adjacent cytosolic compartment and the percentage of BRN3A-positive nuclei that had adjacent mCherry-BCLX_L_ labeling. One section from four transduced eyes was counted.

### Imaging of the ON tract

Euthanized mice were decapitated and the heads were placed in 4% PFA for 24 h. The skullcap and brain were then dissected away, taking care to leave the optic tract intact. Imaging was performed on a Zeiss Discovery V8 Stereo fluorescent microscope (Zeiss, Oberkochen, Germany). Images were taken using the Zen blue software (Zeiss).

### Whole mounting of retinas, imaging, and calculation of nuclear densities

Retinas were whole mounted as previously described [30] using VECTASHIELD Antifade Mounting Medium containing DAPI (Vector Laboratories, Burlingame, CA). Imaging and quantification of cellular density were performed as previously described [30]. Counted nuclei were restricted to cells with nuclear morphology typical of neurons (round euchromatic appearance with prominent nucleoli). At least 29 retinas were counted for each group. Statistical significance was calculated using a one-sided *t* test, which assumed equal variance between groups.

### Quantitative PCR (qPCR)

Retinas that were used for qPCR were flash frozen in microcentrifuge tubes on dry ice. cDNA was created as previously described [30]. qPCR was performed using an ABI Quant Studio 7 RT PCR machine (Thermo Fisher Scientific). Primers for *S16*, *BclX*_*L*_, *Nefl*, *Nrn1*, *Thy1*, *Sncg*, *Tubb3*, *Gfap*, *Hsp27*, and *Gap43* have been described previously [37]. Absolute abundances of each transcript were calculated using a standard curve of *S16* ribosomal protein mRNA [37]. Four groups of three pooled eyes were used for each experimental cohort. Statistical significance between the transcript abundances of different groups were calculated using a one sided *t* test assuming equal variance.

### ON fixation, sectioning, paraphenylenediamine (PPD) staining, and scoring

ONs were processed for PPD staining as previously described [38]. Sections were imaged on a Zeiss Axioimager Z2 upright microscope using a ×40 magnification oil objective. Images were scored using a semi-quantitative three-score system where each nerve was assigned a score of No or Early (NOE), Moderate (MOD), or Severe (SEV) glaucoma [20]. All ONs were scored by two masked observers. The statistical significance between the distributions of different groups was calculated using *χ*^2^ test.

## Results

### mCherry*-*BCLX_L_ prevents GFP-BAX recruitment in vitro

**Fig. 1:**
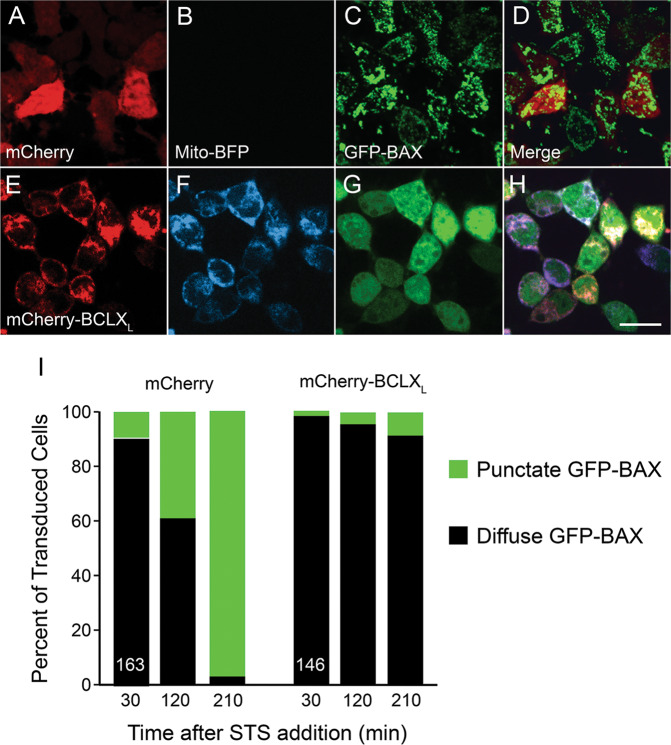
mCherry-BCLX_L_ colocalizes with the mitochondria and prevents BAX recruitment in vitro. Representative images of D407 cells expressing mCherry (**A**–**D**), mCherry-BCLX_L_ (**E**–**H**) along with mitoBFP and GFP-BAX 3.5 h after treatment with 1 µM staurosporine (STS). Note that mitoBFP fluorescence is rapidly lost when the mitochondrial outer membrane becomes permeabilized and the mitochondria become fragmented, which is why **B** has dramatically reduced fluorescence. Scale bar = 10 µm. Live cell imaging was used to track the localization of GFP-BAX for 3.5 h after STS treatment. The percentage of cells in each condition with punctate localization is quantified in **I**. *N* = 163 and 146 cells for the mCherry and mCherry-BCLX_L_ expressing groups, respectively. *χ*^2^ test was used to assess the significance of the difference in percentage between each group at each time point. *P* < 0.0005 at 30 min, *P* < 0.00001 at 120 min, and *P* < 0.00001 at 210 min.

The canonical function of mCherry*-*BCLX_L_ of preventing BAX from accumulating on the mitochondria was verified in vitro. As expected, mCherry-BCLX_L_ colocalized with a mitochondrially localized BFP (Fig. 1E, F, H). The ability of mCherry-BCLX_L_ to prevent GFP-BAX translocation was assessed in cells after STS treatment. Over 95% of cells expressing mCherry and GFP-BAX exhibited GFP-BAX translocation to mitochondria within 3.5 h (Fig. 1A–D, I). Conversely, only 8% of cells expressing mCherry-BCLX_L_ and GFP-BAX exhibited punctate GFP-BAX (Fig. 1E–I).

### *AAV2-Pgk-mCherry-BclX*_*L*_ efficiently transduces RGCs

**Fig. 2:**
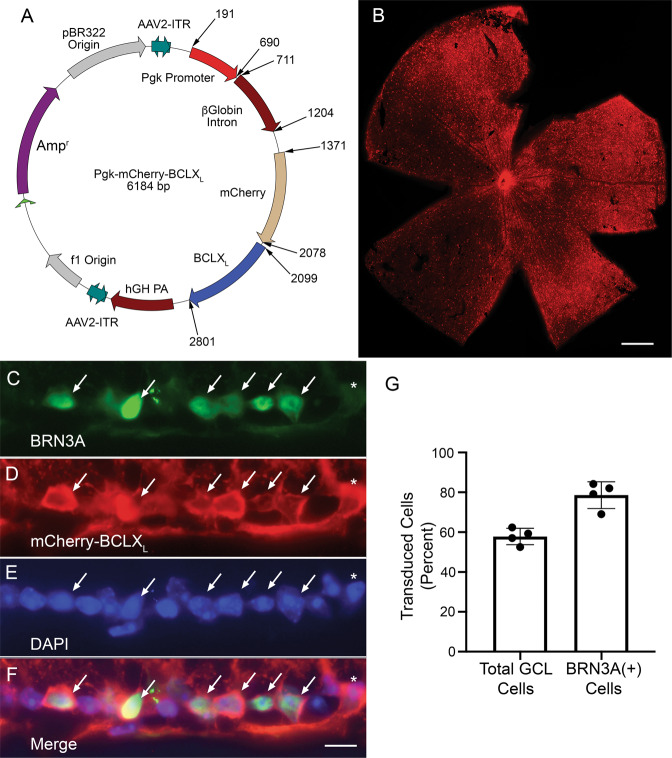
The intraretinal expression pattern of mCherry-BCLX_L_ following *AAV2-Pgk-mCherry-BclXL* transduction. **A** A map showing the plasmid that was packaged into *AAV2-Pgk-mCherry-BclX*_*L*_. **B** A retinal whole-mount preparation, 4 weeks after intravitreal injection, showing wide-spread expression of the transgene across the retina. Scale bar = 450 µm. **C**–**F** Co-localization of BRN3A-positive cells that were transduced by *AAV2-Pgk-mCherry-BclX*_*L*_ (arrows). An asterisk labels a transgene expressing cell that is BRN3A negative but appears to project a labeled axon into the nerve fiber layer. Only the ganglion cell layer (GCL) is shown. Scale bar = 20 µm. **G** Graph showing the percentages of total cells in the GCL and BRN3A-positive cells that expressed the mCherry-BCLX_L_ transgene (mean ± standard deviation).

Next, the transduction efficiency of *AAV2-Pgk-mCherry-BclX*_*L*_ (Fig. 2A) was measured. Evaluation of retinal whole mounts 4 weeks after intravitreal injection showed wide-spread expression of the transgene in the ganglion cell layer (Fig. 2B). Histologic examination of transduced retinas showed that *AAV2-Pgk-mCherry-BclX*_*L*_ transduced over 50% of the cells in the ganglion cell layer of the retina, which is consistent with the percentage of RGCs in this layer [39]. Colocalization with the RGC marker BRN3A demonstrated that nearly 80% of BRN3A+ cells were transduced (Fig. 2C–G). AAV2 also transduces other retinal cell types [31]. We observed transduction of both horizontal and amacrine cells based on colocalization with an antibody to Calbindin [40] (Fig. S1).

**Fig. 3:**
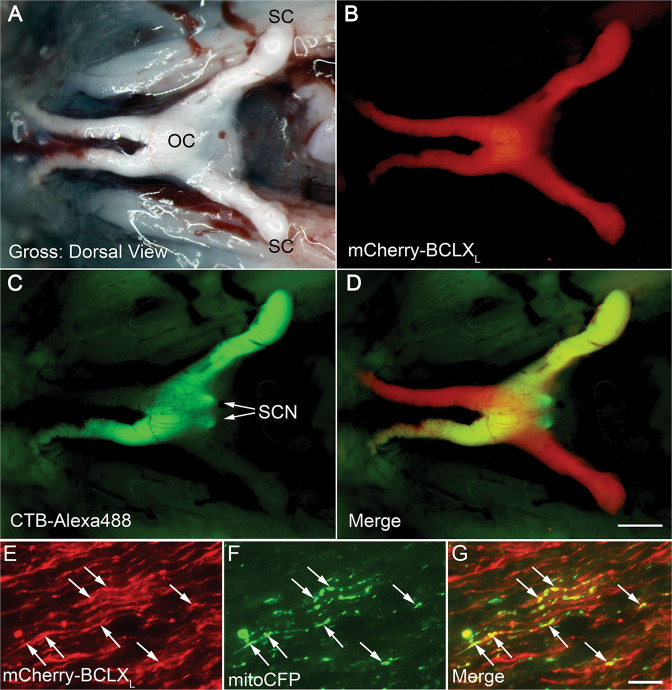
mCherry-BCLXL is robustly expressed in the RGC axons in the optic nerve. **A** A brightfield image of the dorsal view of the optic nerves from a mouse transduced bilaterally with AAV2-Pgk-mCherry-BCLX_L_ and later injected unilaterally (left eye only) with CTB-Alexa488. **B** The same field imaged for mCherry-BCLX_L_ fluorescence. The fusion protein can be seen in the optic nerve distal to the optic chiasm (OC) and approaching the superior colliculus (SC). **C** The same field imaged for CTB-Alexa488 fluorescence. Arrows denote fluorescence in the suprachiasmatic nucleus (SCN). **D** A merged image of **B**, **C** showing bilateral mCherry-BCLX_L_ expression and unilateral CTB-488. Scale bar = 1 mm. **E**–**G** Histologic sections of the optic nerve from a transduced eye of a transgenic mouse expressing *Thy1-mitoCFP* to label RGC mitochondria. Note that not all RGCs express *Thy1-mitoCFP* in this line. The mCherry-BCLX_L_ transgene is present throughout individual axons but concentrated in regions that correspond to axonal mitochondria (arrows). Scale bar = 5 µm.

The mCherry-BCLX_L_ fusion protein was also observed throughout the optic tract of transduced eyes, demonstrating its presence in RGC axons (Fig. 3). Unilateral injection of CTB-488, to label axons, in a mouse that was bilaterally injected with *AAV2-Pgk-mCherry-BclX*_*L*_ showed colocalization with the fusion protein, confirming axonal labeling. CTB-488 also showed bilateral fluorescence in the suprachiasmatic nucleus (SCN; Fig. 3C arrows), the only region of the rodent brain that is innervated equally on the ipsilateral and contralateral sides by projections from one ON [41], principally from intrinsically photosensitive RGCs [42]. No mCherry-BCLX_L_ fluorescence was detected in the SCN, suggesting that *AAV2-Pgk-mCherry-BclX*_*L*_ did not transduce this population of RGCs. Transgenic mice expressing *Thy1-mitoCFP* to label RGC mitochondria were also injected. ON sections showed colocalization of mCherry-BCLX_L_ and mitochondria in RGC axons (Fig. 3E–G).

### mCherry*-*BCLX_L_ prevents cell loss in RGC layer following ONC

**Fig. 4:**
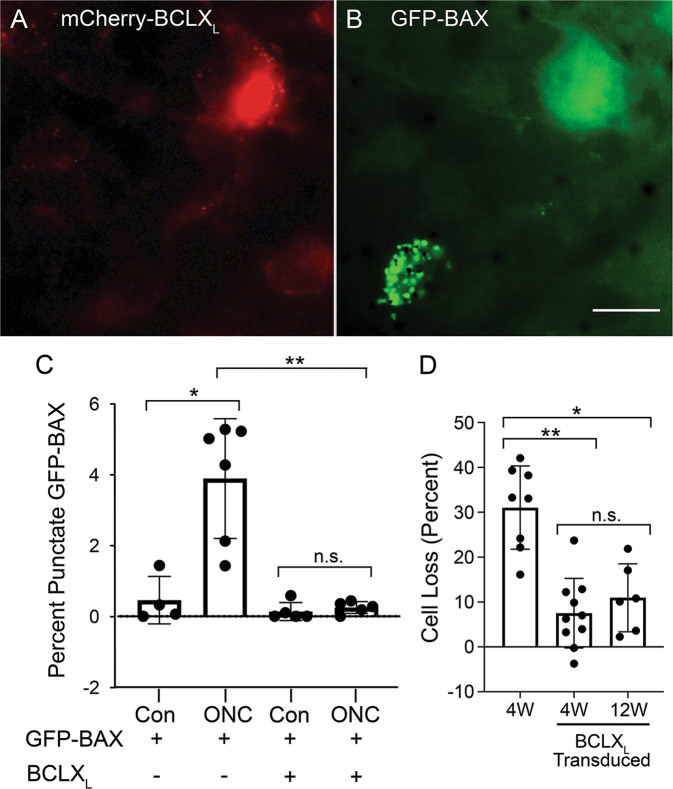
mCherry-BCLX_L_ expression prevents BAX translocation and attenuates cell loss after optic nerve crush. **A**, **B** Panels showing mCherry-BCLX_L_ and GFP-BAX expression, respectively, in a retinal whole mount from a mouse that that had undergone ONC to induce BAX activation. The cell expressing mCherry-BCLX_L_ shows diffuse localization of GFP-BAX and the cell that is not expressing mCherry-BCLX_L_ has punctate, translocated GFP-BAX. Scale bar = 10 µm. **C** A quantification of the phenomenon displayed in **A**, **B**. In this experiment, all mice were transduced with *AAV2-Pgk-GFP-BAX*. Half the mice also received an injection of *AAV2-Pgk-mCherry-BCLX*_*L*_. The percentage of GFP-BAX-labeled cells with punctate GFP-BAX was quantified 1 week after ONC in untransduced mice and mice that had been bilaterally transduced with *AAV2-Pgk-mCherry-BclX*_*L*_ 1 month before ONC (mean ± standard deviation). Significance was determined using a one-sided *t* test. In all, 4–7 mice were used per group. **D** Cell loss was determined by quantifying the difference in nuclear density in the RGC layer of retinal wholemounts between the experimental and contralateral eye (mean ± standard deviation). In all, 6–10 mice were used per group. n.s. = not significant. *P < 0.05. **P < 0.01.

We tested the ability of mCherry-BCLX_L_ to protect RGCs from apoptosis following ONC (Fig. 4). Mice were co-transduced with AAV2s expressing mCherry-BCLX_L_ or GFP-BAX. Prevention of BAX translocation after ONC was assessed by counting the percentage of cells with punctate GFP-BAX in the presence or absence of mCherry-BCLX_L_ (Fig. 4A, B). One week after ONC, mCherry-BCLX_L_ overexpression prevented a significant change in the percentage of cells with punctate GFP-BAX (Fig. 4C).

Next, we tested the ability of mCherry-BCLX_L_ to provide sustained protection to RGCs by examining the pattern of cell loss in the RGC layer after ONC. Both 4 and 12 weeks post-ONC, *AAV2-Pgk-mCherry-BclX*_*L*_ transduced retinas had significantly less cell loss than untransduced retinas analyzed 4 weeks post-ONC (Fig. 4D).

### AAV2 transduction does not prevent IOP elevation in DBA/2J mice

**Fig. 5:**
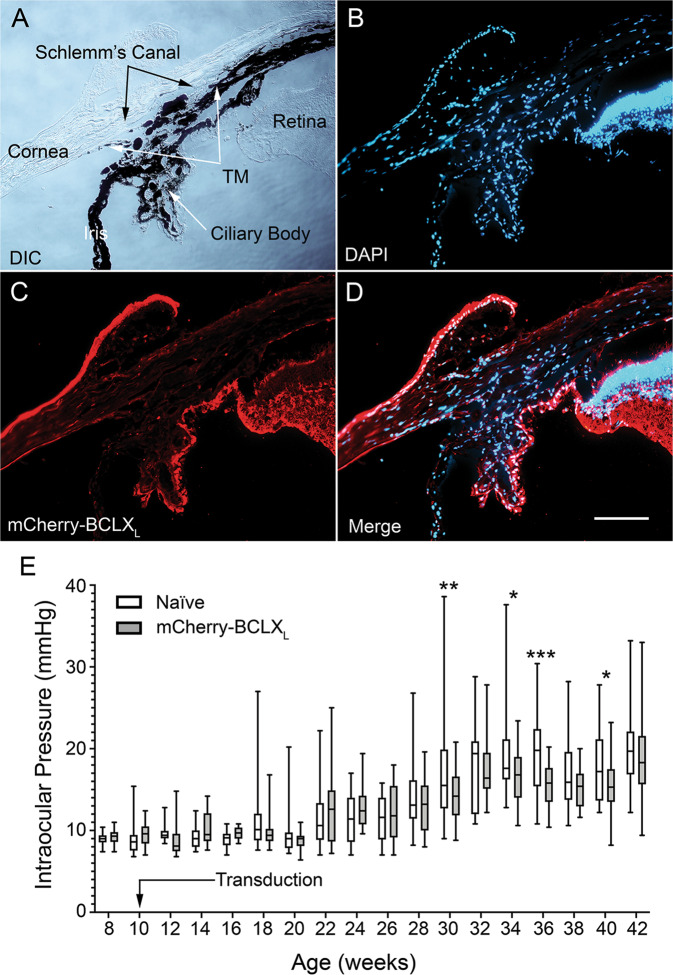
*AAV2-Pgk-mCherry-BclXL* gene therapy does not alter the progression of ocular hypertension in DBA/2J mice. **A** Brightfield image showing the relevant ocular anatomy for the outflow pathway of an eye that was transduced with *AAV2-Pgk-mCherry-BclX*_*L*_. **B**–**D** Fluorescent images showing the DAPI-positive nuclei (**B**) and the expression pattern of mCherry-BCLX_L_ (**C**), as well as a combined view of **B**, **C** (**D**). Note that an mCherry antibody was used to amplify the mCherry-BCLX_L_ signal. Expression is seen strongly in the retina and ciliary body but is not present in Schlemm’s canal, the iris, or the trabecular meshwork (TM). The exterior surface of the cornea also appears to be expressing mCherry-BCLX_L_, which may be the result of viral particles flowing onto the surface of the eye when the needle is removed from the eye during the intravitreal injection. **E** Intraocular pressures of *AAV2-Pgk-mCherry-BclX*_*L*_ transduced and naive DBA/2J mice that were measured every 2 weeks from the age of 8 to 42 weeks. Transduction occurred the day after the second measurement of IOP when the mice were 10 weeks old. The data are represented as box and whisker plots with the mean shown as a bar in the center of the data, the 25th and 75th percentiles shown as the edges of the box, and the whiskers extending to show the minimum and maximum values for the data. For IOP measurements, *N* = an average of 18 eyes per group per time point. Generalized Estimating Equation regression modeling was used to assess the statistical difference between the IOPs of the groups at each timepoint and longitudinally. Individual timepoints that are statistically different from one another are shown. *P < 0.05, **P < 0.01, ***P < 0.001. Regression analysis performed on the data show that the longitudinal IOP progression of the naive and mCherry-BCLX_L_ groups are similar.

Next, we examined whether *AAV2-Pgk-mCherry-BclX*_*L*_ affected the progression of ocular hypertension in DBA/2J mice. Histological examination of transduced cells in the conventional aqueous outflow pathway showed that mCherry-BCLX_L_ was expressed in the non-pigmented epithelial cells of the ciliary body but not in cells of the trabecular meshwork or Schlemm’s canal (Fig. 5A–D). Longitudinal measurements of IOP in *AAV2-Pgk-mCherry-BclX*_*L*_ transduced mice, compared to untransduced mice, revealed four individual timepoints with statistically significant differences in IOP between groups (Fig. 5E). Regression modeling failed to detect a difference between the trends of the groups over the entire course of the experiment, indicating that mCherry-BCLX_L_ did not significantly impact longitudinal IOP elevation.

### mCherry-BCLX_L_ prevents RGC degeneration in aged DBA/2J mice

**Fig. 6:**
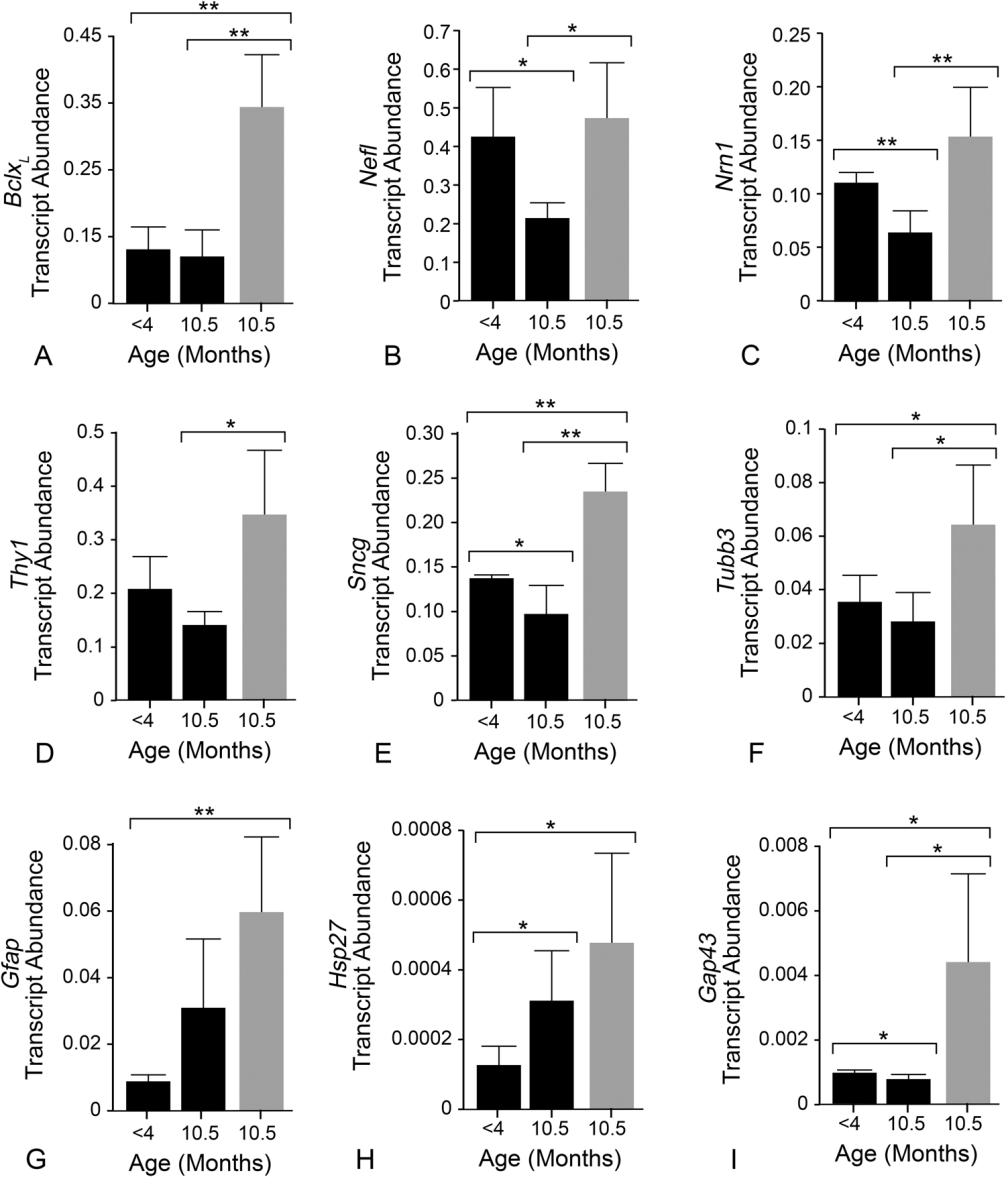
BCLX_L_ gene therapy attenuates RGC-specific transcript depletion in aged DBA/2J mice. Quantitative (real time) PCR was used to assess retinal gene expression in young mice and glaucomatous mice. **A** *BclX*_*L*_ expression was five times higher in transduced, aged DBA/2J mice (grey bars) than either young or 10.5-month-old naive animals (black bars). **B**–**F** The transcript abundances of five RGC-specific/selective transcripts. RGC transcripts were consistently more abundant in aged mice that were BCLX_L_ treated than age-matched mice that did not receive BCLX_L_. **G**, **H** The transcript abundance of *Gfap* and *Hsp27*, two transcripts produced in retinas experiencing glaucomatous stress. These transcripts were significantly more abundant in aged mice relative to the young mice that had not developed glaucoma. **I** The transcript abundance of *Gap43*, a gene that has been associated with neuroregeneration. *Gap43* was more abundant in BCLX_L_-treated, 10.5-month-old mice than young or 10.5-month-old naive mice. Four groups of three pooled eyes were used for each experimental cohort. The graphs display the average abundance of the four groups and the error bars show the standard deviation from that average. All transcript levels are quantified as the number of target molecules present per molecule of *S16* ribosomal protein mRNA. Statistical evaluation was made using unpaired *t* tests of the means (*P < 0.05, **P < 0.01).

Next, we tested whether mCherry-BCLX_L_ would prevent RGC degeneration in aged DBA/2J mice. Retinal whole mounts showed robust expression of the transgene in transduced retinas at both 10.5 and 12 months of age (Fig. S2). The protective effect of mCherry-BCLX_L_ was first measured by quantifying changes in the abundance of transcripts selectively expressed in RGCs using qPCR. At 10.5 months of age, BCLX_L_ treated retinas exhibited a nearly 5-fold increase in *BclX*_*L*_ transcript abundance (Fig. 6A). These retinas also had significantly higher abundances of the RGC-selective mRNAs *Nefl*, *Nrn1*, *Thy1*, *Sncg*, and *Tubb3* transcripts than untreated retinas, which showed a nearly uniform decrease in these mRNAs (Fig. 6B–F), indicating that BCLX_L_ treatment preserved RGC-specific gene expression. Both naive and treated retinas exhibited similar increases in *Gfap* and *Hsp27* transcript abundance relative to young mice, indicating that BCLX_L_ treatment did not prevent retinal stress (Fig. 6G, H). Interestingly, treated retinas expressed more *Gap43* (Fig. 6I), a marker of axon regeneration [43].

**Fig. 7:**
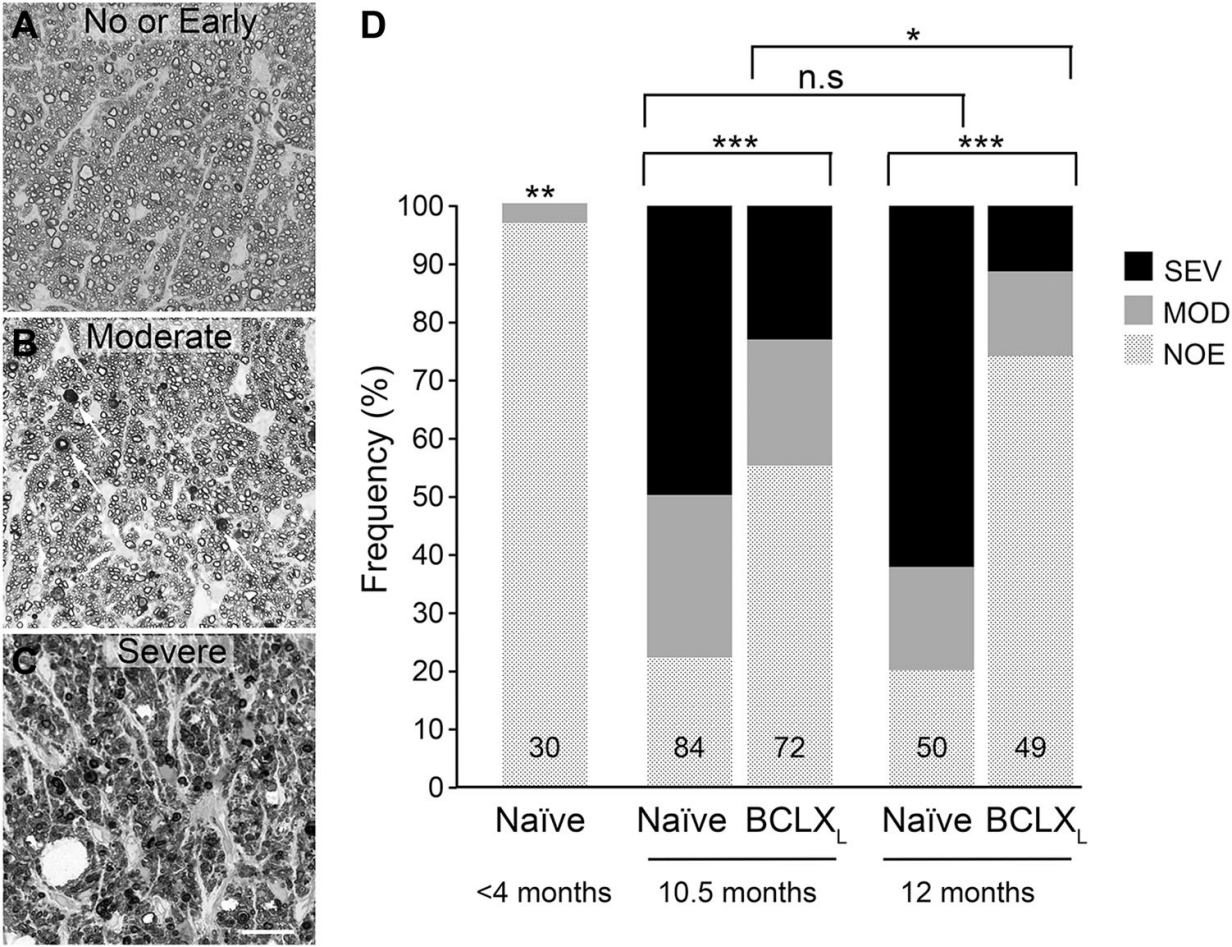
mCherry-BCLX_L_ gene therapy prevents optic nerve degeneration in aged DBA/2J mice. **A**–**C** Examples of each potential optic nerve score. **A** The no or early (NOE) glaucoma example has uniform distribution of axons and the glial cells occupy minimal space on the image. **B** The moderate glaucoma (MOD) example has a large number of healthy axons but also displays increased dark spots (denoted by white arrows) where the myelin sheaths have collapsed around the axons. The glial cells may have become slightly enlarged but do not occupy much more space than the NOE example. **C** The severe glaucoma (SEV) example has very few healthy axons, most of what remains are dark spots. The glial cells in this image have expanded and occupy a larger percentage of the image than either the NOE or moderate glaucoma examples. Scale bar = 25 µm. **D** Distributions of each of the three scores for each experimental group. The inset number denotes the number of optic nerves scored for each group. The distribution of mice for each cohort (M/F) was: <4 (4/12); 10.5 Naive (21/21); 10.5 BCLX_L_ (22/15); 12 Naive (13/12); 12 BCLX_L_ (11/14). *χ*^2^ tests were performed to assess the statistical differences between the distributions of scores of different groups. *P < 0.01, **P < 0.00003 (young mice relative to all other cohorts in individual tests), ***P < 0.00001.

**Fig. 8:**
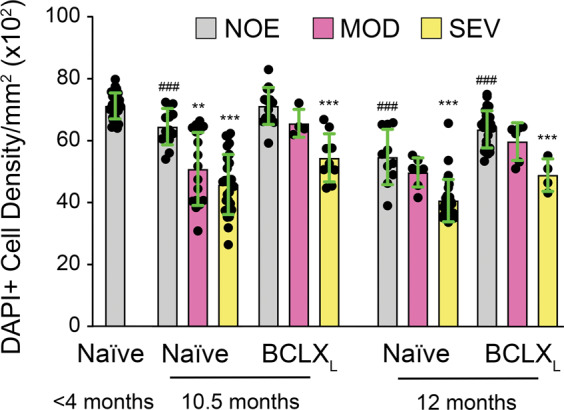
Correlation between retinal total cell density and optic nerve degeneration score. Eyes for which there was both an optic nerve score and analysis of the total neuronal cell density present in the retina were used to evaluate the symmetry of both RGC soma loss and axon loss in the same eye. Total cell counts reflect both RGC and amacrine neuronal populations in the ganglion cell layer. Combined scatter plot/bar graphs (mean ± standard deviation) are shown. In all cohorts (naive and BCLX_L_ treated, aged 10.5 or 12 months), cell density was significantly lower in eyes with SEV optic nerves compared to eyes with NOE optic nerves in the same cohort (Student’s *t* test, ***P < 0.0001). Also, within cohorts, eyes exhibiting MOD optic nerves had no significant change in cell density, compared to eyes with NOE optic nerves, with the exception of naive mice aged to 10.5 months (**P = 0.0006). Compared to young naive mouse eyes with NOE optic nerves, most aged cohorts had modestly reduced cell densities in the NOE populations (on average a 10% reduction, ^###^*P* < 0.001), with the exception of BCLX_L_-treated eyes at 10.5 months, which showed no significant change. The distribution of mice for each cohort (M/F) was: <4 (6/10); 10.5 Naive (17/12); 10.5 BCLX_L_ (10/5); 12 Naive (12/11); 12 BCLX_L_ (8/12).

BCLX_L_ gene therapy prevented glaucomatous degeneration. BCLX_L_-treated mice had significantly fewer moderately and severely degenerated ONs compared to naive animals at 10.5 months of age (Fig. 7). BCLX_L_ gene therapy was profoundly protective of axons in the ONs of 12-month-old animals, an age when *Bax* deficiency was not protective [6]. In fact, the distribution of ON scores for 12-month-old BCLX_L_-treated mice showed modestly, but significantly, less degeneration than the distribution of scores for 10.5-month-old BCLX_L_-treated mice. Stratification of data where we had both cell density counts and ON scores for the same eye showed that there was an association with reduced cell density and SEV ON score, while eyes exhibiting MOD ON damage typically showed no significant loss of cell density compared to eyes with NOE damage within a given cohort (Fig. 8). We interpret the presence of SEV damage and cell loss in some mice from treated cohorts as a consequence of less-than-optimal viral transduction, although other factors such as extreme ocular hypertension cannot be ruled out. Additionally, cell density in retinas from eyes with NOE ON damage, in most cohorts of aged mice, still exhibited an overall 10% decline in total cell density when compared with young mice, suggesting age-related effects not affected by the gene therapy.

These data reflect the protective effect of BCLX_L_ therapy if applied prophylactically (4–5 months). To test whether we can achieve similar protection during the period of first onset of elevated IOP, mice were transduced at 7 months and aged to 10.5 months. These mice exhibited a similar level of protection compared to mice transduced at an earlier age (Fig. S3).

## Discussion

The mCherry-BCLX_L_ fusion protein prevented BAX translocation both in vitro and in vivo (in RGCs) after exposure to acute apoptotic stimuli. In the DBA/2J mouse model of glaucoma, mCherry-BCLX_L_ conferred extended protection to the retina and the ON that exceeded the reported protective effect of genetic deletion of *Bax*. Therefore, BCLX_L_ must protect RGCs through more than simple inhibition of BAX.

There are several potential explanations for the additional protection conferred by mCherry-BCLX_L_. Recent literature has focused on the axodegenerative pathway catalyzed by SARM1 [5, 44, 45]. A combination of the *Wld*^*s*^ allele, which prevents SARM1 activation, and depletion of BAX, via genetic deletion of one *Bax* allele, appeared to provide a greater protective effect to ONs of DBA/2J mice than either treatment alone [3]. Our results suggest that BCLX_L_ is able to inhibit both the BAX and SARM1 pathways. SARM1 activation is the result of an increased ratio of nicotinamide mononucleotide (NMN) to NAD^+^ [46]. Future studies should examine whether BCLX_L_ stabilizes the ratio of NMN:NAD^+^ or acts by some indirect mechanism, such as increasing the resiliency of the mitochondria, consistent with growing evidence that an age-related decline in mitochondrial performance is a contributing factor to glaucomatous neurodegeneration [25, 47]. Interestingly, BCLX_L_ has been shown to improve the metabolic efficiency of mitochondria and increase overall mitochondrial biomass [48–50]. Future studies should explore how BCLX_L_ augments mitochondrial function in RGCs.

A final possibility is that BCLX_L_ helps the RGC maintain axonal transport. Axonal transport disruption is one of the earliest events in glaucoma [51]. The sustained protection in glaucomatous animals by our fusion protein suggests that mCherry-BCLX_L_ was present in axons at high enough concentrations throughout the course of the disease to counteract degenerative signaling. A by-product of putative augmentation of mitochondrial physiology by BCLX_L_ may be uninterrupted axonal transport.

BCLX_L_ gene therapy yielded better preservation of ONs in 12-month-old DBA/2J mice than in 10.5-month-old mice. Since the 10.5- and 12-month groups were performed sequentially, this variation may be the result of a stochastic batch effect. However, the increased abundance of RGC-specific transcripts and the regeneration marker *Gap43* suggests some level of spontaneous regeneration occurring in these mice after ocular hypertension subsides, a process which begins between 11 and 12 months of age in these mice [20]. The effect of BCLX_L_ on the regenerative potential of RGCs is a promising, untested future direction.

These results suggest that BCLX_L_ gene therapy preserves RGC anatomy and gene expression in a mouse model of glaucoma. To build on these findings, future studies should test BCLX_L_ gene therapy in a larger animal model of glaucoma. Larger animals more accurately reflect the human anatomy and disease and the potential challenges with gene therapy in human retinas [52]. Additionally, our experiments did not examine the potential to preserve RGC function, which should be a priority in a model system better suited to clinical examination. Translational application of BCLX_L_ gene therapy will also require evaluating the safety issues associated with long-term expression of this anti-apoptotic protein, including its possible effects on tumorigenesis [53, 54] and increased susceptibility to viral infections [55].

## Supplementary information

Supplemental Figure Legends

Figure S1

Figure S2

Figure S3

## Data Availability

Raw data files used for this study will promptly be made available upon reasonable request to the authors. The reagent “AAV2-*Pgk-mCherry-BCLX*_*L*_” is the subject of a provisional patent filed by the Wisconsin Alumni Research Foundation (WARF).
