# Supplementary material for: BCLXL gene therapy moderates neuropathology in the DBA/2J mouse model of inherited glaucoma
Source: Cell Death Dis. 2021 Aug 10;12(8):781. doi: 10.1038/s41419-021-04068-x (PMC8355227; doi:10.1038/s41419-021-04068-x)
Supplement: Supplementary file 1 — Supplemental Figure Legends [file 41419_2021_4068_MOESM1_ESM.docx]

Supplemental Figures

Figure S1. Retinal section showing co-localization of the mCherry-BCLX_L_ transgene and Calbindin.

Intravitreal injection of AAV2-Pgk-mCherrry-BCLX_L_ results in modest transduction of cells in both the inner nuclear layer (INL) and ganglion cell layer (GCL). Counter staining with an antibody to Calbindin indicates that these cells are likely horizontal neurons (arrows) which exhibit weak expression of the transgene, and a population of amacrine cells (arrowheads) which exhibit strong transgene expression. Cells in the GCL that are transduced and co-localize with Calbindin could be either amacrine cells or retinal ganglion cells^55^. No transgene expressing cells in the outer nuclear layer (ONL) were detected. Individual channels are shown. Scale bar = 70 µm.

Figure S2. Whole mounted retinas showing mCherry-BCLX_L_ expression in the ganglion cell layer of DBA/2J mice.

(Top panels) Retina of a 10.5 month old mouse and (Bottom panels) 12 month mouse showing cells with robust expression of the transgene. Individual channels are shown. Scale bar = 20 µm.

Figure S3. Glaucomatous damage scores in 10.5 month old DBA/2J mice transduced at 7 months of age. A) Histographs of optic nerve scores. The data shown for young naïve mice (<4 months old), naïve mice at 10.5 months, and mice at 10.5 months treated with *AAV2/2-Pgk-mCherry-BCLX_L_* at 4-5 months of age are reproduced from Figure 7. These data are compared to 10.5 month old DBA/2J mice transduced with virus at 7 months of age. Statistical comparisons are only shown for the 7 month treated group relative to the other cohorts (statistics for the other groups are shown in Figure 7). The distribution of mice for each cohort (M/F) was: <4 (4/12); 10.5 Naïve (21/21); 10.5 (transduced at 4-5 mo.) BCLX_L_ (22/15); 10.5 (transduced at 7 mo.) BCLX_L_ (12/16). χ^2^ tests, n.s.=not significant, ***P<0.0001. B) Scatter plot showing total retinal cell density as a function of optic nerve score. The data for <4 month old mice is reproduced from Figure 8. The distribution of mice transduced at 7 months was 10 male and 16 female. Student *t*-tests, *P=0.0035, **P=0.001.
