# Supplementary figures and images for: BCLXL gene therapy moderates neuropathology in the DBA/2J mouse model of inherited glaucoma
Source: Cell Death Dis. 2021 Aug 10;12(8):781. doi: 10.1038/s41419-021-04068-x (PMC8355227; doi:10.1038/s41419-021-04068-x)

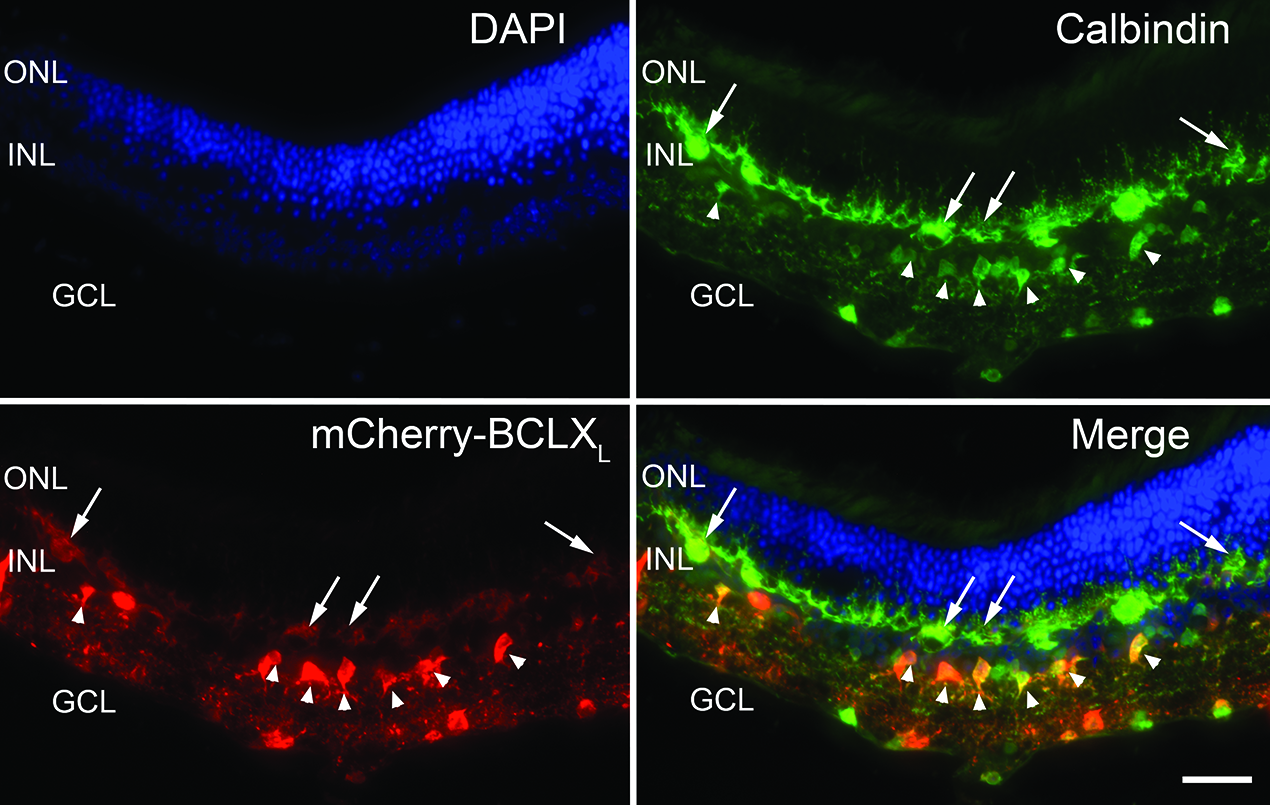

Supplement: Supplementary file 2 — Figure S1 [file 41419_2021_4068_MOESM2_ESM.tif]

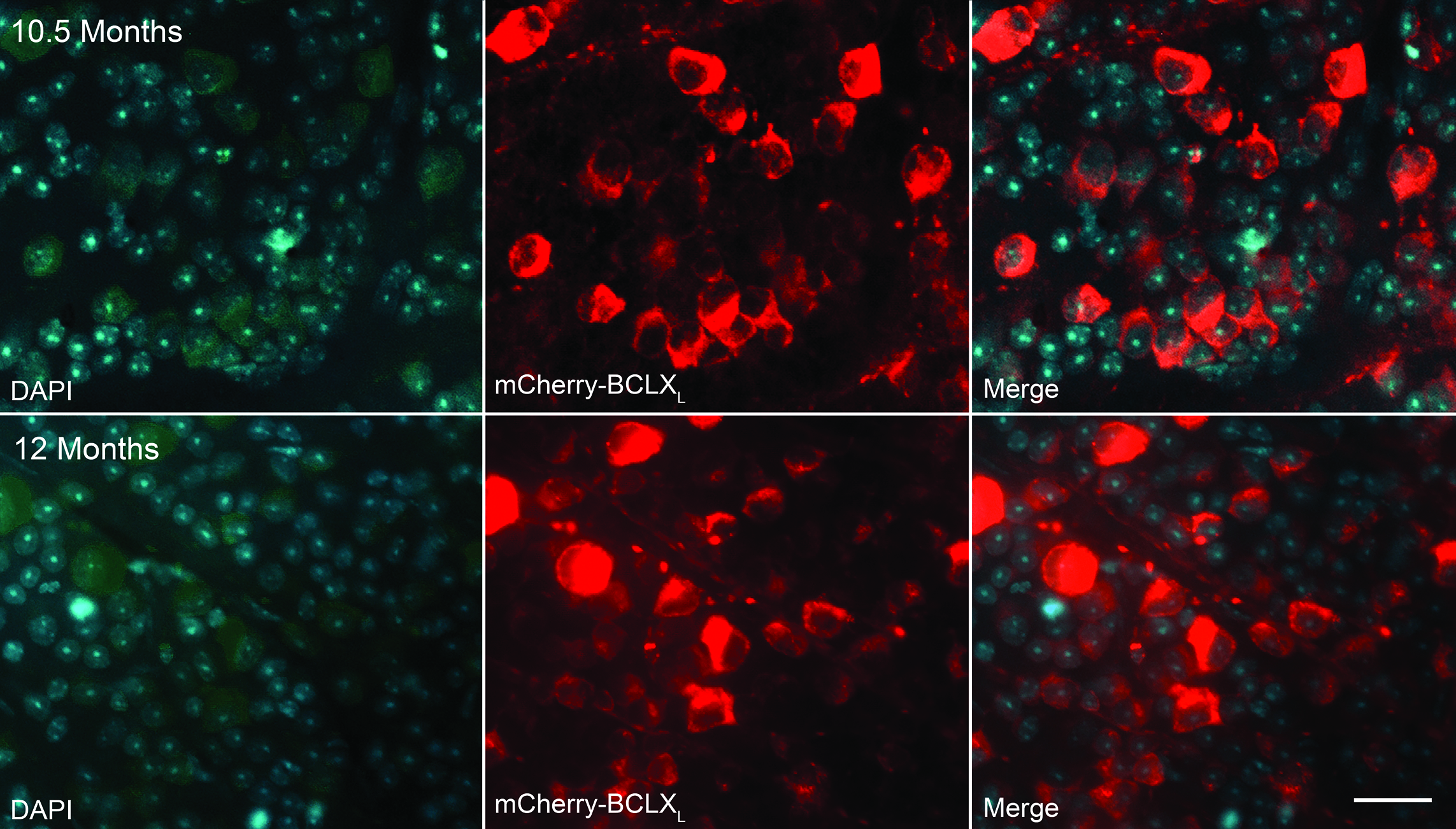

Supplement: Supplementary file 3 — Figure S2 [file 41419_2021_4068_MOESM3_ESM.tif]
